# Loss of Down syndrome critical region-1 leads to cholesterol metabolic dysfunction that exaggerates hypercholesterolemia in ApoE-null background

**DOI:** 10.1016/j.jbc.2021.100697

**Published:** 2021-04-23

**Authors:** Masashi Muramatsu, Tsuyoshi Osawa, Yuri Miyamura, Suguru Nakagawa, Toshiya Tanaka, Tatsuhiko Kodama, Hiroyuki Aburatani, Juro Sakai, Sandra Ryeom, Takashi Minami

**Affiliations:** 1 Division of Molecular and Vascular Biology, IRDA, Kumamoto University, Kumamoto, Japan; 2 Division of Integrative Nutriomics, The University of Tokyo, Tokyo, Japan; 3 Division of Genome Science, The University of Tokyo, Tokyo, Japan; 4 Division of Systems Biology, The University of Tokyo, Tokyo, Japan; 5 Division of Metabolic Medicine, RCAST, The University of Tokyo, Tokyo, Japan; 6 Division of Molecular Physiology and Metabolism, Graduate School of Medicine, Tohoku University, Sendai, Japan; 7 Department of Cancer Biology, University of Pennsylvania, Philadelphia, Pennsylvania, USA

**Keywords:** hypercholesterolemia, apolipoprotein (Apo)E, Down syndrome critical region (DSCR)-1, nuclear factor for activated T cells (NFAT), proprotein convertase subtilisin/kexin type (PCSK)9, low-density lipoprotein (LDL), nonalcoholic fatty liver disease (NAFLD), regulator of calcineurin (RCAN)1, Apo, apolipoprotein, DSCR, Down syndrome critical region, ER, endoplasmic reticulum, GO, gene ontology, HEK, human embryonic kidney, HFD, high-fat diet, HRP, horseradish peroxidase, LDL, low-density lipoprotein, LDLR, LDL receptor, NAFLD, nonalcoholic fatty liver disease, NFAT, nuclear factor for activated T cell, oxLDL, oxidative LDL, PCSK, proprotein convertase subtilisin/kexin, SREBP, sterol regulatory element-binding protein, VEGF, vascular endothelial growth factor, WT, wild-type

## Abstract

Down syndrome critical region (DSCR)-1 functions as a feedback modulator for calcineurin-nuclear factor for activated T cell (NFAT) signals, which are crucial for cell proliferation and inflammation. Stable expression of DSCR-1 inhibits pathological angiogenesis and septic inflammation. DSCR-1 also plays a critical role in vascular wall remodeling associated with aneurysm development that occurs primarily in smooth muscle cells. Besides, *Dscr-1* deficiency promotes the M1-to M2-like phenotypic switch in macrophages, which correlates to the reduction of denatured cholesterol uptakes. However, the distinct roles of DSCR-1 in cholesterol and lipid metabolism are not well understood. Here, we show that loss of *apolipoprotein (Apo) E* in mice with chronic hypercholesterolemia induced *Dscr-1* expression in the liver and aortic atheroma. In *Dscr-1*-null mice fed a high-fat diet, oxidative- and endoplasmic reticulum (ER) stress was induced, and sterol regulatory element-binding protein (SREBP) 2 production in hepatocytes was stimulated. This exaggerated ApoE^−/−^-mediated nonalcoholic fatty liver disease (NAFLD) and subsequent hypercholesterolemia. Genome-wide screening revealed that loss of both *ApoE* and *Dscr-1* resulted in the induction of immune- and leukocyte activation-related genes in the liver compared with *ApoE* deficiency alone. However, expressions of inflammation-activated markers and levels of monocyte adhesion were suspended upon induction of the *Dscr*-1 null background in the aortic endothelium. Collectively, our study shows that the combined loss of Dscr-1 and ApoE causes metabolic dysfunction in the liver but reduces atherosclerotic plaques, thereby leading to a dramatic increase in serum cholesterol and the formation of sporadic vasculopathy.

Atherosclerosis is one of the major causes of mortality worldwide due to the severe complications associated with it, such as acute cerebral and myocardial infarctions. A clinical manifestation of whole-body cholesterol metabolism is an age-related increase in the plasma levels of low-density lipoprotein (LDL) cholesterol (1). This increase in LDL, referred to as hypercholesterolemia, has a significant impact on cardiovascular disease risk because of the insufficient removal of LDL from circulation, which is mediated in part by the endothelial cell response to denatured, oxidized LDL. Apolipoprotein (Apo) E- or LDL receptor-deficient mouse models have been used extensively to study vascular inflammation leading to atherosclerosis. Moreover, proprotein convertase subtilisin/kexin type 9 (PCSK) binds to the LDL receptors (LDLR) and accelerates their degradation. PCSK9 has been explored as an attractive therapeutic target for hypercholesterolemia and atherosclerosis (2).

The endothelium constitutes a continuous cellular lining of vascular networks and is an important locus for critical regulatory nodes to maintain homeostatic balance. Endothelial cell activation, if persistent or spatially and temporally misplaced, can lead to vasculopathy. Extracellular mediators, such as vascular endothelial growth factor (VEGF) and thrombin, promote key regulatory pathways for endothelial cell proliferation, inflammation, and angiogenesis (3, 4). Tight control of these signaling pathways is essential for maintaining vascular homeostasis. Thus, understanding the molecular mechanisms and signaling pathways underlying vascular diseases can help identify new therapeutic targets.

Epidemiological studies have revealed that individuals with Down syndrome have lower than expected rates of atherosclerotic disease and hypertension. The Down syndrome critical region (DSCR)-1 gene (also known as RCAN1, MCIP-1, calcipressin-1, or Adapt78) is a feedback modulator of the VEGF-calcineurin nuclear factor for activated T cell (NFAT) pathway (5, 6). DSCR-1 also functions to mitigate oxidative stress (7, 8), indicating its crucial role in regulating endothelial cell activation and maintaining vascular homeostasis (5). More recently, it has been shown that the Dscr-1^−/−^ mutation alters the population of macrophage subtypes; M1 and M2 subsets dampen ApoE^−/−^-mediated atherosclerotic progression in mice (9).

Sterol regulatory element-binding protein (SREBP) 1 and 2 are transcription factors that regulate the expression of genes involved in lipid and cholesterol synthesis, respectively. Both transcription factors function as key nodes of convergence and divergence within biological signaling networks relating to a variety of pathophysiological processes (10, 11). SREBP2 can activate a myriad of cellular processes and pathologies, such as reactive oxygen species production, endoplasmic reticulum (ER) stress, and apoptosis. However, the underlying molecular mechanisms are complex and require further investigation to fully understand (12).

In the present study, we examined lipid metabolic dysfunction associated with DSCR-1 and SREBP2 signaling. *Dscr-1* null mice are predisposed to nonalcoholic fatty liver disease (NAFLD). In an atherosclerotic ApoE^−/−^ background or PCSK9 overexpression, *Dscr-1* null mice revealed significant hypercholesterolemia leading to lipid accumulation in the peripheral tissues. Our findings will potentially lead to new ways to protect against vascular diseases, such as hypercholesterolemia, by exploiting the antioxidative and anti-ER stresses capacities mediated by DSCR-1.

## Results

### DSCR-1 protects the liver from oxidative stress and SREBP2 hyperactivation

**Figure 1:**
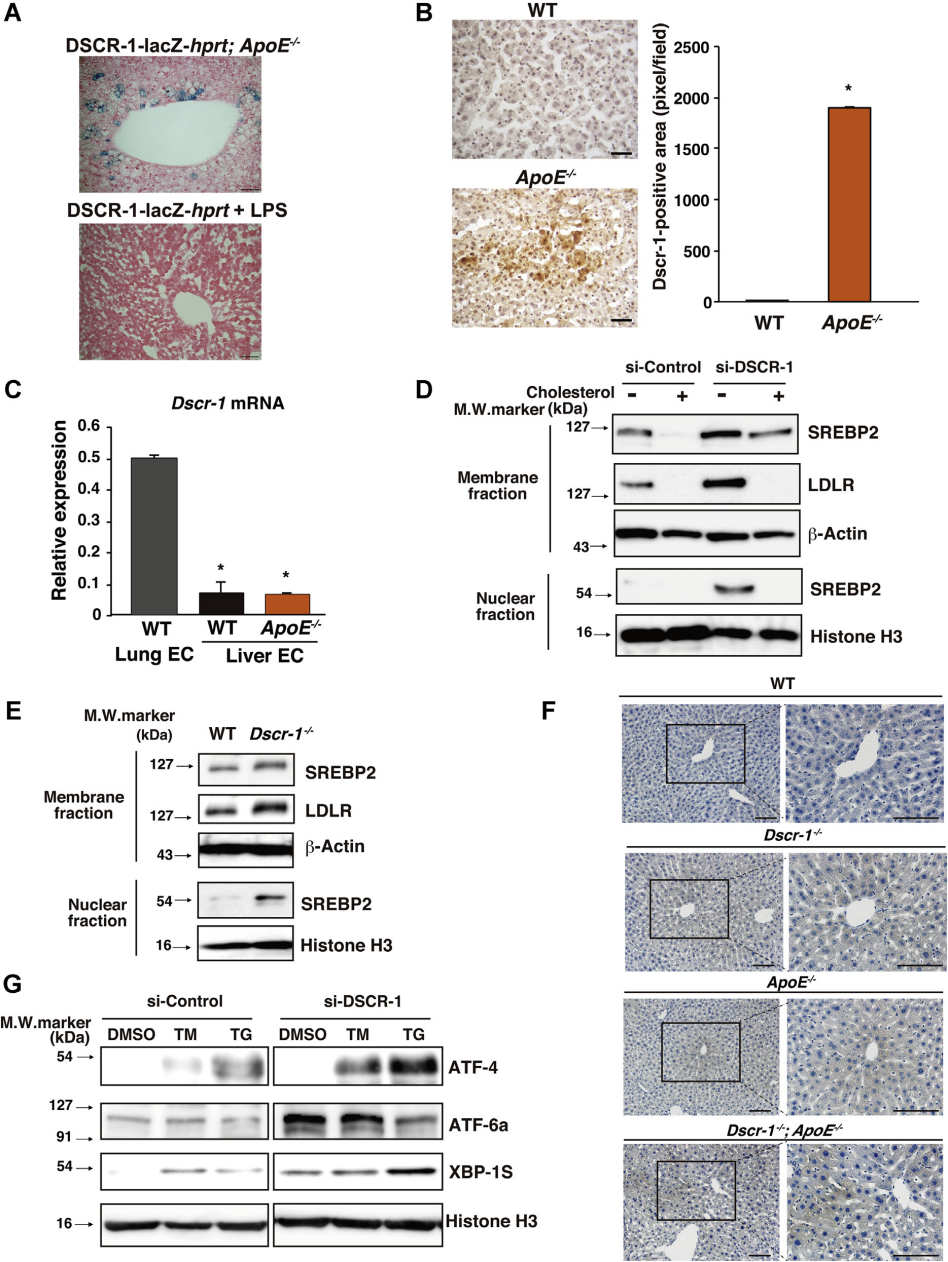
**Dscr-1 deficiency induces oxidative and ER stress and SREBP2 hyperactivation in the liver.***A*, X-gal staining in the liver from DSCR-1-*lac*Z-hprt reporter mice crossed with ApoE^−/−^ (*upper*) or treated with LPS for 24 h (*lower*). *B*, representative liver sections of immunohistochemical staining using anti-Dscr-1 antibody from WT and ApoE^−/−^ mice. *Brown color* indicates Dscr-1-positive hepatocyte with hematoxylin counterstaining. Scale bar: 50 μm. *Right bar graph* shows quantitative analysis of Dscr-1-positive area (n = 6, mean ± SD) ∗*p* < 0.01 compared with WT. *C*, real-time qPCR analysis of mouse Dscr-1 short variant mRNA from lung or liver endothelium (n = 3, mean ± SD). ∗*p* < 0.001 compared with endothelial cells from lung in WT mice. *D* and *E*, western blots of HepG2 cells treated with si-DSCR-1 compared with si-control in the presence or absence of cholesterol-starved medium (*D*). Western blots of primary cultured hepatocytes from WT or Dscr-1^−/−^ mice (*E*). Membrane/cytosol fraction or nuclear fraction incubated with anti-SREBP2 or -LDLR antibodies. β-Actin or histone H3 shown as the loading control. Data are representative of three independent experiments. *F*, immunostaining of 4-HNE (indicated in *brown*) in liver sections from WT, Dscr-1^−/−^, ApoE^−/−^, or combined loss of Dscr-1 plus ApoE mice. Low magnification (*left*) and high magnification image (*right*) from each *rectangle* are shown, derived from four independent optimal fields in three independent experiments. Scale bar; 100 μm. *G*, western blots of HepG2 cells treated with si-DSCR-1 compared with si-control and with tunicamycin (TM), thapsigargin (TG), or mock control (DMSO). ER stress markers ATF-4, ATF-6a, and XBP-1S are also shown. Histone H3 indicates the loading control. Data shown are representative of three independent experiments.

The calcineurin-NFAT-DSCR-1 signaling axis is crucial in vascular activation and is involved in inflammation and angiogenesis (5). We demonstrated that DSCR-1 is specifically expressed in the endothelium during embryo development (13). In adults, DSCR-1 is upregulated in vasculopathy regions, such as the tumor endothelium, cloudy cornea, and septic inflamed endothelial cells in the heart and lungs (8, 13). To analyze this further, we investigated whether Dscr-1 expression could serve as a marker for dysfunctional vasculature due to chronic inflammation. We crossed our transgenic reporter mice with the *DSCR*-1 promoter driving *LacZ* with an ApoE^−/−^ background, and the mice developed spontaneous atherosclerotic plaques at about 32 weeks of age. Significant *Dscr-1* promoter activation was not observed in most ApoE^−/−^ mice organs, except for a subset of hepatocytes that demonstrated lacZ-positive staining (Fig. 1*A*, *upper* column). Significant DSCR-1 protein expression was also detected in hepatocytes (Fig. 1*B*). However, DSCR-1 expression was not induced in sinusoid endothelial cells of the ApoE null mutation; the level was very low compared with that in lung endothelial cells (Fig. 1*C*). Moreover, consistent with previous findings (13), acute septic inflammation failed to activate DSCR-1 expression in the liver (Fig. 1*A*, *lower* column). First, we determined the functional role of DSCR-1 in cholesterol metabolism. SREBP2 is a known key transcriptional regulator of cholesterol biosynthesis and uptake into cells (12). To test whether SREBP2 activity varies under DSCR-1 expression in the liver, we conducted western blotting with anti-SREBP2 antibodies in cultured hepatocytes (HepG2) in the presence or absence of siRNA against DSCR-1 (si-DSCR-1). With si-DSCR-1 treatment, expression of the full-length precursor form of SREBP2 was increased in cholesterol-deprived media compared with that in cholesterol-containing media. Truncated mature active SREBP2 was detected only with si-DSCR-1 treatment. Following expression of active SREBP2 levels, downstream LDLR expression was increased in hepatocytes (Fig. 1*D*). Similarly, the DSCR-1 null mutation resulted in a significant induction in both precursor and mature forms of SREBP2, resulting in more LDLR in the primary liver of the mice (Fig. 1*E*). DSCR-1 knockdown possibly activates NFAT (14). NFAT1 overactivation can induce SREBP2 promoter activity, which was dampened in the presence of the NFAT inhibitor cyclosporine A (CsA) (Fig. S1*A*).

In many cases, cholesterol metabolic dysfunction is related to oxidative or ER stress in hepatocytes (15, 16). We recently reported that DSCR-1 protects against oxidative LDL-mediated inflammation and pathological angiogenesis in microvascular endothelial cells (8). To verify this in the liver microenvironment, tissue sections were stained with 4-hydroxy-2-nonenal (HNE) as an oxidative stress marker. As shown in Figure 1*F*, stress signals (indicated in brown) were significantly increased in Dscr-1^−/−^ and ApoE^−/−^ mice hepatocytes. Interestingly, such oxidative stress markers were more profoundly stimulated by the combined null mutation of *Dscr-1* and *ApoE* than either alone. Next, to test whether ER stress is also associated with si-DSCR-1, we performed western blotting with three major ER stress sensors: PKR-like ER kinase (PERK)-mediated ATF4, inositol requiring (IRE) 1-mediated XBP1s, and activating transcription factor (ATF) 6-mediated ATF6a. When HepG2 cells were treated with tunicamycin or thapsigargin, the expression of ATF-4 and XBP1s, but not ATF-6a, was induced, and expression was further stimulated in the presence of si-DSCR-1 (Fig. 1*G*). Taken together, these findings suggest that DSCR-1 upregulation in hepatocytes is not just a “warning” signal, but it also actively functions in protection from oxidative stress and/or ER stress. The lack of DSCR-1 exacerbates these stresses, resulting in a promotion of cholesterol synthesis *via* the crucial transcription factor SREBP2.

### DSCR-1 protects the liver from inflammation-mediated cholesterol metabolic dysfunction

**Figure 2:**
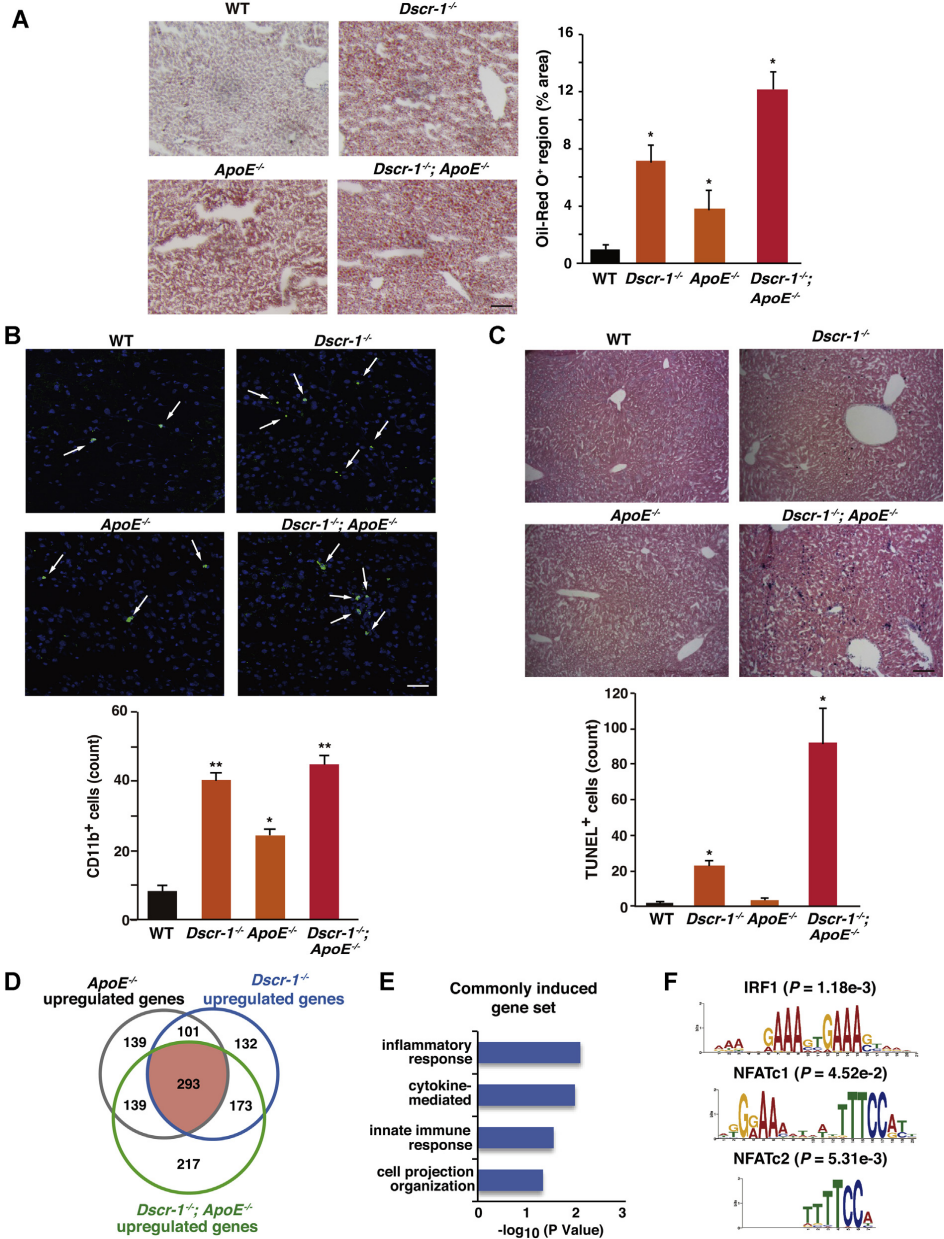
**Dscr-1 deficiency promotes the development of NAFLD and inflammation in the liver.***A*, Oil-Red O staining of liver sections from each HFD fed conditioned mouse. Scale bar: 100 μm. Oil-red O positive regions were quantified as % area shown in *right bar graph* (n = 6, mean ± SD). ∗*p* < 0.05 compared with WT. *B*, CD11b^+^ cell-infiltrated regions (shown by *white arrows*) in each liver after HFD were stained with anti-CD11b antibody (*green*) and DAPI (*blue*). CD11b^+^ cells were counted from each optical field (n = 6, mean ± SD), shown in the *bottom bar graph*. ∗*p* < 0.05 and ∗∗*p* < 0.01 compared with WT. *C*, TUNEL assays in each liver sample after HFD for 8 weeks. TUNEL^+^ cells are indicated with *blue color*. Cell volumes were quantified in each optimal field (n = 6, mean ± SD) shown in the *bottom bar graph*. ∗*p* < 0.05 compared with WT. *D*, Venn diagrams of more than twofold upregulated gene volume in the liver from two independent microarrays of each conditioned mouse. *E*, GO analysis of commonly upregulated gene set from *red color* in panel *D*. *F*, MEME-ChIP analysis with upstream 1000 bp promoter region of commonly upregulated genes in panel *D*. *p*-values indicating probability were calculated using TOMTOM.

To uncover the effect of *DSCR-1* loss-induced dysregulation in hepatocytes, leading to liver dysfunction, especially in high-fat diet (HFD) conditions, mice with null mutations of *Dscr-1*, *ApoE*, and the combination were fed HFD for 12 weeks. As shown in Figure 2*A*, ApoE^−/−^ mice demonstrated lipid deposition, indicating metabolic dysfunction. More importantly, after being fed HFD, more severe NAFLD-like phenotypes were observed in the livers of *Dscr-1* single mutant and *Dscr-1* plus *ApoE* double null mutant mice. These results suggest that *Dscr-1* protects against ApoE^−/−^-mediated lipid metabolic changes responsible for chronic liver dysfunction. To investigate the mechanism underlying the defect in cholesterol clearance in *ApoE* and *Dscr-1* double null mutant mice, liver sections were immunohistochemically stained. Since DSCR-1 acts as a feedback modulator for proinflammatory NFAT signaling (17, 18), *Dscr-1* null mice were predicted to be susceptible to proinflammatory microenvironments (13). Consistent with this hypothesis, Dscr-1^−/−^ mice fed with HFD showed markedly enhanced CD11b^+^ leukocyte infiltration in the liver (Fig. 2*B*). Notably, HFD-fed ApoE^−/−^ mice livers also exhibited inflammatory leukocyte infiltration, albeit at lower levels than in Dscr-1^−/−^ mice (Fig. 2*B*). Liver damage caused by HFD induced apoptosis in a subset of liver cells in *Dscr-1*^*−/−*^ and *Dscr1*^−/−^ plus *ApoE*^−/−^ mice, whereas *ApoE*^−/−^ mice experienced no significant apoptosis in the liver. The number of TdT-mediated dUTP nick end labeling (TUNEL)-positive cells was significantly high in the livers of Dscr-1^−/−^ mice and even higher in the livers of *Dscr1*^−/−^ and *ApoE*^−/−^ mice (Fig. 2*C*). Moreover, to analyze the genetic changes in the liver microenvironment, microarrays were performed in each genotype of mice fed normal chow (Fig. 2*D* and Fig. S1*B*). Gene set enrichment analysis (GSEA) revealed that homeostasis and cell-proliferation-regulated genes were enriched in the livers of *ApoE*, *Dscr-1*, and combined null mutant mice, respectively (Fig. 2*E* and Fig. S1, *C*–*E*). Based on Gene Ontology (GO) analysis, 293 genes associated with inflammation, cytokine signaling, and immune responses were commonly upregulated in *Dscr-1* and *ApoE* double null mice (Fig. 2*E*). Interestingly, commonly upregulated gene sets were regulated by the NFAT transcription factor family and interferon response factor (IRF)-1 (Fig. 2*F*).

Taken together, these data suggest that defects in cholesterol transfer observed in *ApoE*^−/−^ mice are due, in part, to NFAT-mediated inflammation in hepatocytes, as shown by *Dscr-1* promoter activation (Fig. 1*A*). In turn, *Dscr-1* null mice on HFD developed steatohepatitis due to oxidative or ER stress upregulation and SREBP2 hyperactivation. Thus, the combined loss of *Dscr-1* and *ApoE* indicated synergistically exaggerated liver-damage-mediated dysregulation compared with just *ApoE* loss.

### Dscr-1^−/−^ and ApoE^−/−^ double knockout mice exhibit high-cholesterol lipid profiles

**Figure 3:**
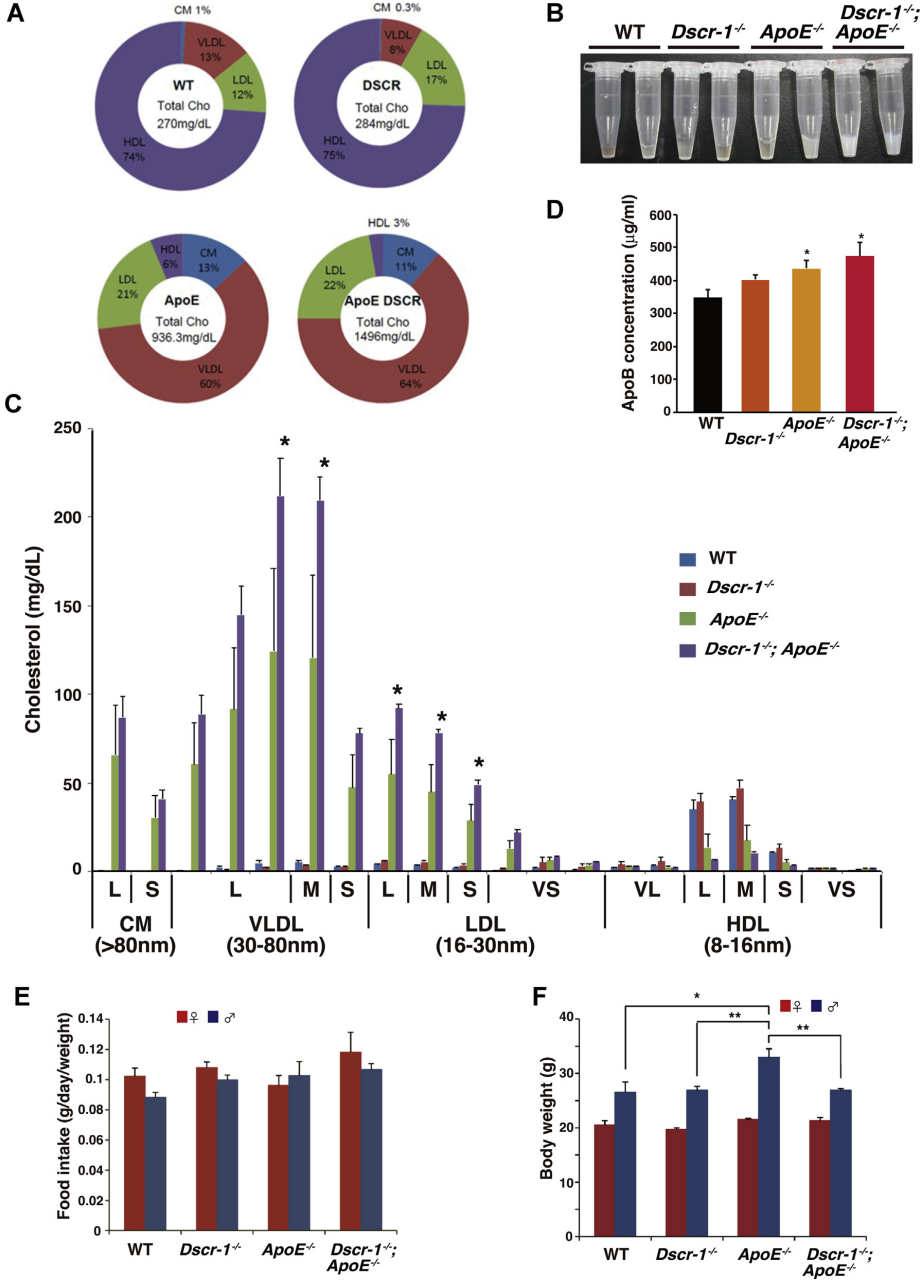
**Dscr-1 deficiency exaggerates hypercholesterolemia by ApoE**^**−/−**^**condition.***A*, pie chart of total cholesterol volume and their proportions (%). Values are derived from six independent specimens. *B*, two representative collected serum samples from each conditioned mouse are shown. *C*, cholesterol content profiles from each conditioned mouse (n = 12, mean ± SD) are shown. ∗*p* < 0.05 compared with ApoE^−/−^ and Dscr-1^−/−^ plus ApoE^−/−^. *D*, ELISA analysis for plasma ApoB quantification from WT, Dscr-1^−/−^, ApoE^−/−^ and double null mutant mice (n = 3, mean ± SD). ∗*p* < 0.05 compared with WT. *E* and *F*, food intake (*E*) and total body weight (*F*) from either or combined null mutation of Dscr-1 and ApoE and littermate control (WT) after an HFD feeding for 8 weeks (n = 12). *Red* and *blue* indicate female and male, respectively (n = 12, mean ± SD). ∗*p* < 0.05 and ∗∗*p* < 0.01 compared to WT. L, large; M, middle; S, small; VL, very large; VS, very small size of lipid.

To analyze whether chronic liver dysfunction in Dscr-1^−/−^ or ApoE^−/−^ mice affected blood lipoprotein profiles, serum was collected from each genotype after HFD treatment. Consistent with previous findings, ApoE^−/−^ mice exhibited increased levels of very-low-density lipoprotein (VLDL) and the remnants, as well as reduced high-density lipoprotein (HDL) levels compared with wild-type (WT) littermate controls (19) (Fig. 3*A*). Notably, mice with combined *Dscr-1* and *ApoE* deletions showed about 1.6-fold higher total cholesterol levels than *ApoE* single knockout mice (Fig. 3, *A* and *B*). Dscr-1^−/−^ mice had a cholesterol profile similar to that of WT controls, whereas ApoE^−/−^ mice showed an increase in VLDL and LDL, which was statistically significantly increased when combined with a *Dscr-1* null mutation (Fig. 3*C*). To further analyze whether the loss of *Dscr-1* leads to an increase in cholesterol production, ApoB lipoprotein levels were quantified in the plasma. Compared with WT controls, Dscr-1^−/−^ mice plasma did not show a significant increase in ApoB, whereas ApoE^−/−^ mice plasma demonstrated an increase in remnant lipoprotein levels that were eventually reflected in ApoB increase (Fig. 3*D*), suggesting that increased hypercholesterolemia in *Dscr1*^−/−^ and *ApoE*^−/−^ double mutant mice is a result of exaggerated ApoE^−/−^-mediated impairment of cholesterol clearance. Moreover, there were no significant differences in terms of food intake level and total weight between the mutant and WT mice (Fig. 3, *E* and *F*). In contrast, ApoE^−/−^ mice showed significantly increased total triglyceride levels, but no further increase was observed with the loss of *Dscr-1* (Fig. S2). Collectively, these findings suggest that ApoE^−/−^ mediated hypercholesterolemia was exaggerated by the null mutation of *Dscr-1* due to the impairment of cholesterol clearance.

### LDLR knockdown exhibits high-cholesterol lipid profiles under *Dscr-1* null mutation

**Figure 4:**
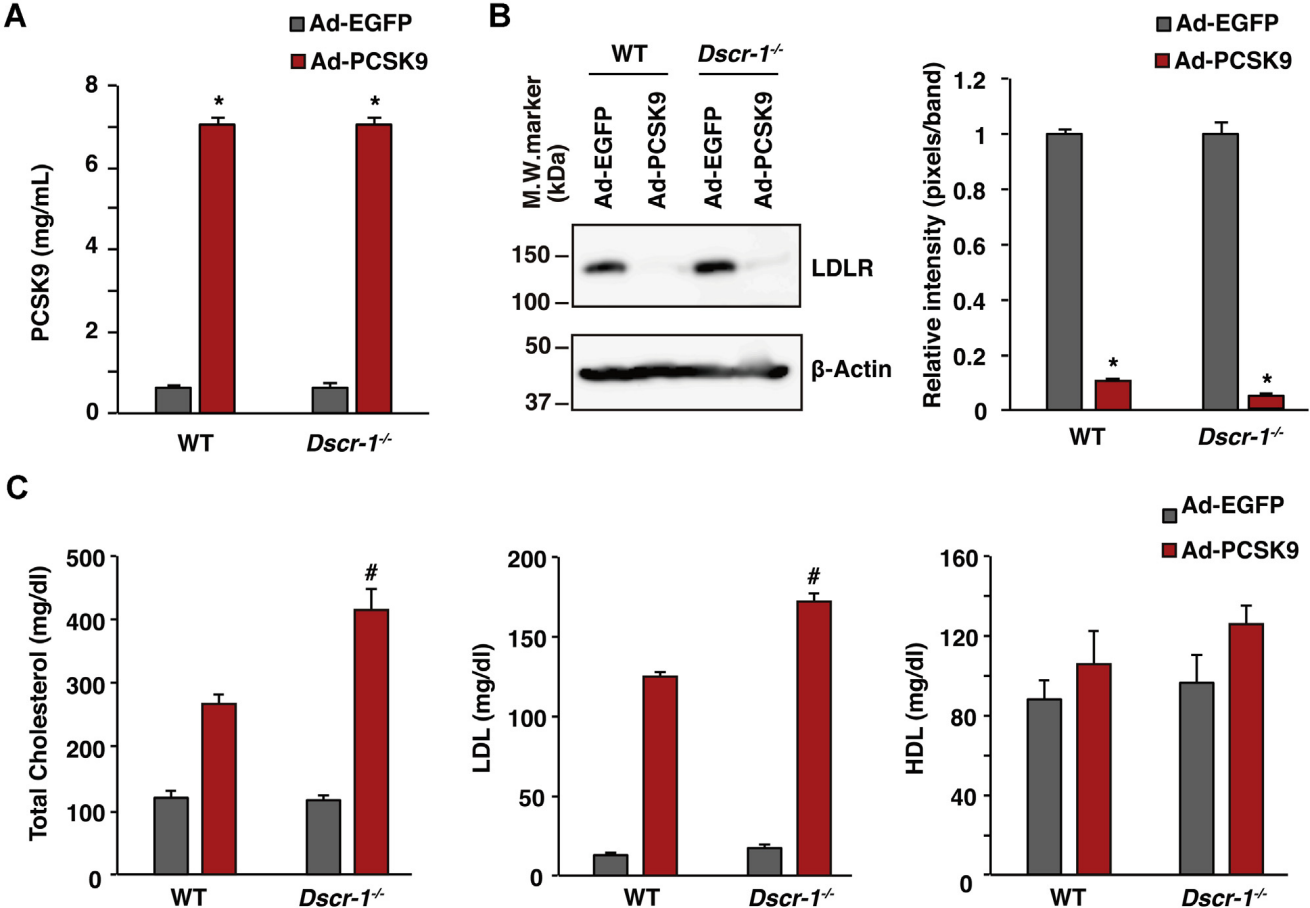
**Dscr-1 deficiency exaggerates hypercholesterolemia under LDLR knockdown conditions.***A*, serum PCSK9 levels 7 days after adenovirus administration. Ad-EGFP was used as the control (n = 3, mean ± SD). ∗*p* < 0.05 compared with control. *B*, western blots of anti-LDLR antibody from total liver extracts. β-Actin indicates the loading control. *Right bar graph*; quantification of LDLR expression from western blots (*left*) (n = 3, mean ± SD). ∗*p* < 0.05 compared with control. *C*, quantification of total cholesterol (*left*), LDL (*middle*), and HDL (*right*) from indicated conditions (n = 3, mean ± SD). #*p* < 0.05 compared with the CA-PCSK9 treatment from WT control.

Subsequently, we examined whether the pathological exacerbation of *ApoE* deficiency is due to direct hypercholesterolemia or indirectly mediated by loss of the *ApoE* gene, since APOE has been reported to possess several pleiotropic effects that influence cell signaling (20). Therefore, we conducted a cholesterol profiling assay under LDLR knockdown conditions. When we used the constitutively activated (CA)-PCSK9 administration method to promote LDLR degradation in the liver of mice, mimicking LDLR knockout mice, loss of DSCR-1 alone failed to increase endogenous autocleavage of PCSK9. In contrast, adenoviral-overexpressed CA-PCSK9 increased the enzyme level in serum from both WT and Dscr-1^−/−^ mice (Fig. 4*A*), eventually resulting in the degradation of LDLR expression in the liver; no obvious difference in LDLR degradation was observed between WT and Dscr-1^−/−^ mice (Fig. 4*B*). Importantly, total cholesterol and LDL levels were significantly higher in Dscr-1^−/−^ mice than in the WT control, whereas HDL levels did not significantly change in either mouse after CA-PCSK9 treatment (Fig. 4*C*). Similarly, hypercholesterolemia *via* LDL receptor knockdown was exaggerated in Dscr-1^−/−^ mice after adeno-associated virus-mediated CA-PCSK9 treatment (Fig. S3). Collectively, these data suggest that the severe pathology observed in *Dscr-1* and *ApoE* double null mutant mice is based on advanced hypercholesterolemia rather than the indirect effects of *ApoE* loss.

### Increased cholesterol circulation in peripheral tissues of Dscr-1^−/−^ and ApoE^−/−^ mice

**Figure 5:**
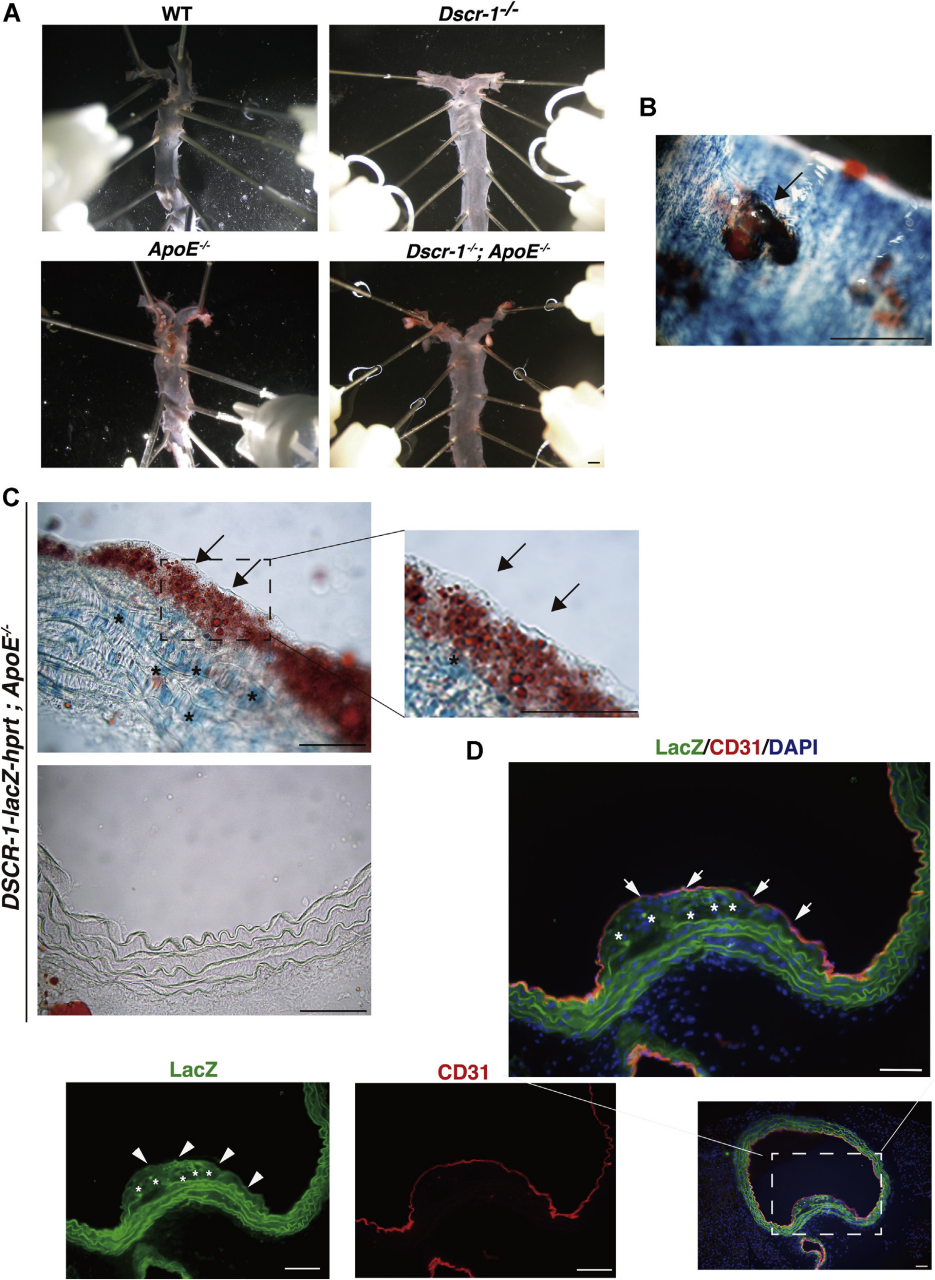
**Dscr-1 expresses atherosclerotic plaques.***A*, macroscopic view of representative Oil-Red O staining with dissected aorta from WT, Dscr-1^−/−^, ApoE^−/−^, and Dscr-1^−/−^ plus ApoE^−/−^ mice. Scale bar: 2 mm. *B*, whole-mount lacZ staining of atherosclerotic plaques in the aorta of DSCR-1 promoter-lacZ-*hprt* locus knock-in mice under ApoE^−/−^ genetic background. *Arrow* indicates representative atheroma. Scale bar: 100 μm. *C*, section images from the aorta. *Lac*Z-positive endothelial cell layer and smooth muscle cells are indicated by *arrows* and *asterisks*, respectively. A high-power view in the plaque field is shown on the *right*. *Upper*, plaque area; *lower*, nonplaque area. *D*, immunostaining of aorta with anti-*Lac*Z (*green*), CD31 (*red*) antibodies. Merged image with DAPI (*blue*) in *rectangle* is magnified. *Lac*Z-positive endothelial cell layer and smooth muscle cells are indicated by *arrows* and *asterisks*, respectively. Data shown are representative of four independent staining experiments. Scale bar: 100 μm.

**Figure 6:**
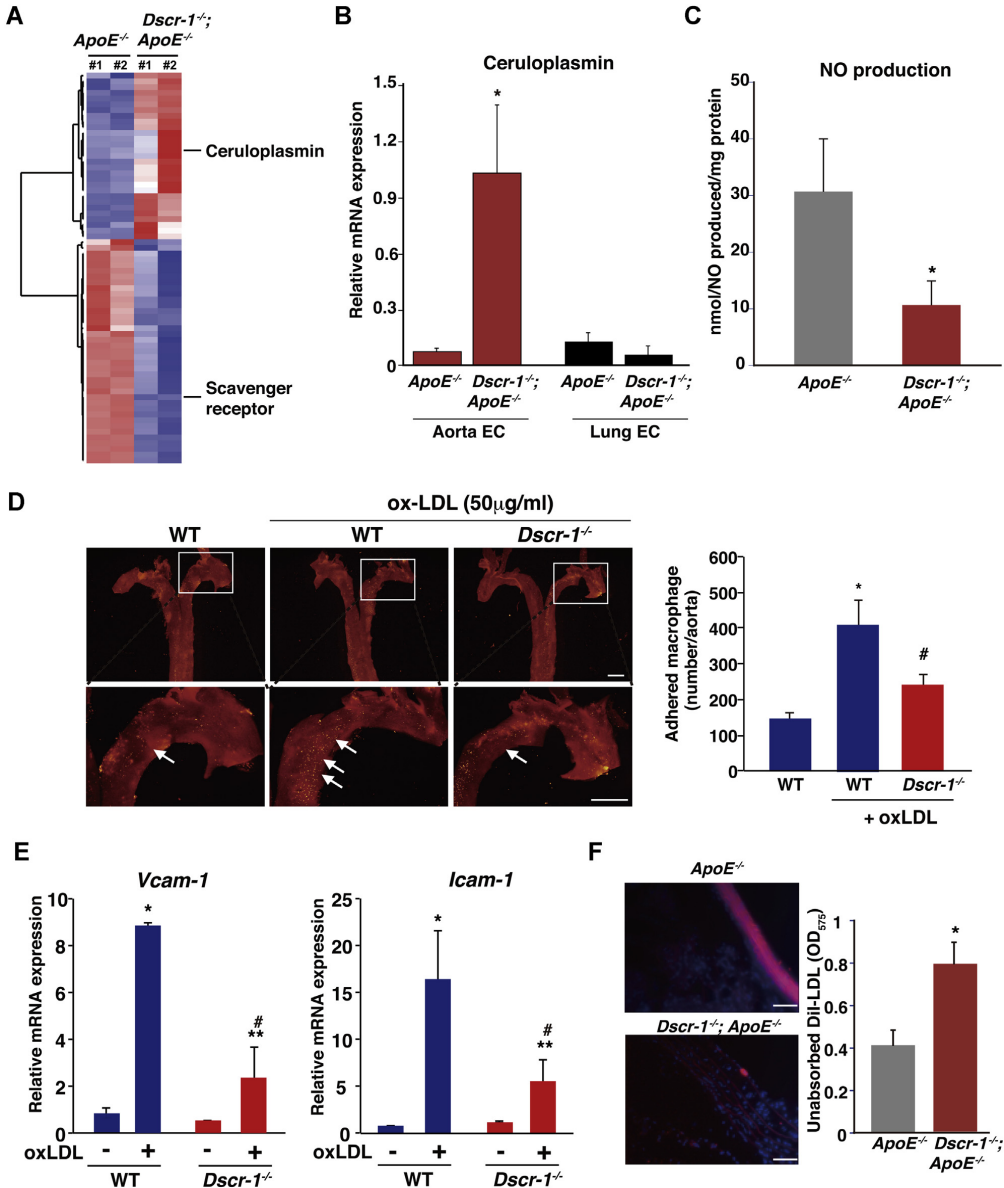
**Dscr-1 deficiency impairs lipid resorption and macrophage adhesion molecule induction in aortic endothelium.***A*, heatmap illustration of up- or down-regulated genes in aortic endothelium from ApoE^−/−^ and Dscr-1^−/−^ plus ApoE^−/−^ mice. *B*, real-time qPCR analysis of ceruloplasmin mRNA expression in indicated endothelial cells. Expression is normalized to cyclophilin A mRNA levels (n = 3, mean ± SD). ∗*p* < 0.05 compared with ApoE^−/−^ in aortic endothelium. *C*, calculated nitric oxide (NO) production levels in each aorta (n = 6, mean ± SD). ∗*p* < 0.05 compared with ApoE^−/−^. *D*, representative images from PKH-26-stained macrophage adhesion (*yellow* in each *rectangle*) from *ex vivo* aorta in the presence or absence of 50 μg/ml oxLDL treatment for 4 h. The *upper rectangular region* is shown with the high-power view of each in the *lower panel*. The *white arrow* indicates a macrophage adhered region. *Right bar graph*; quantitative analysis of adhered macrophage counts (n = 5, mean ± SD) ∗ and #*p* < 0.05 compared with WT in the absence or presence of oxLDL, respectively. Scale bar: 1 mm. *E*, real-time qPCR analysis of *Vcam-1* (*left*) or *Icam-1* (*right*) mRNA expression in aortic endothelium from WT and Dscr-1^−/−^ (n = 5, mean ± SD) ∗*p* < 0.01 and ∗∗*p* < 0.05 compared with the absence of oxLDL, and #*p* < 0.05 compared with WT in the presence of oxLDL. *F*, DiI-labeled LDL (*red*) intake was visualized under fluorescent microscopy. Representative sections from the aorta in ApoE^−/−^ (*upper*) and Dscr-1^−/−^ plus ApoE^−/−^ mice (*lower*) are shown (n = 6). Scale bar: 500 μm. *Right bar graph*; systemic circulated DiI-LDL levels from ApoE^−/−^ and Dscr-1^−/−^ plus ApoE^−/−^ mice (n = 6, mean ± SD) ∗*p* < 0.05 compared with ApoE^−/−^. EC, endothelial cells.

**Figure 7:**
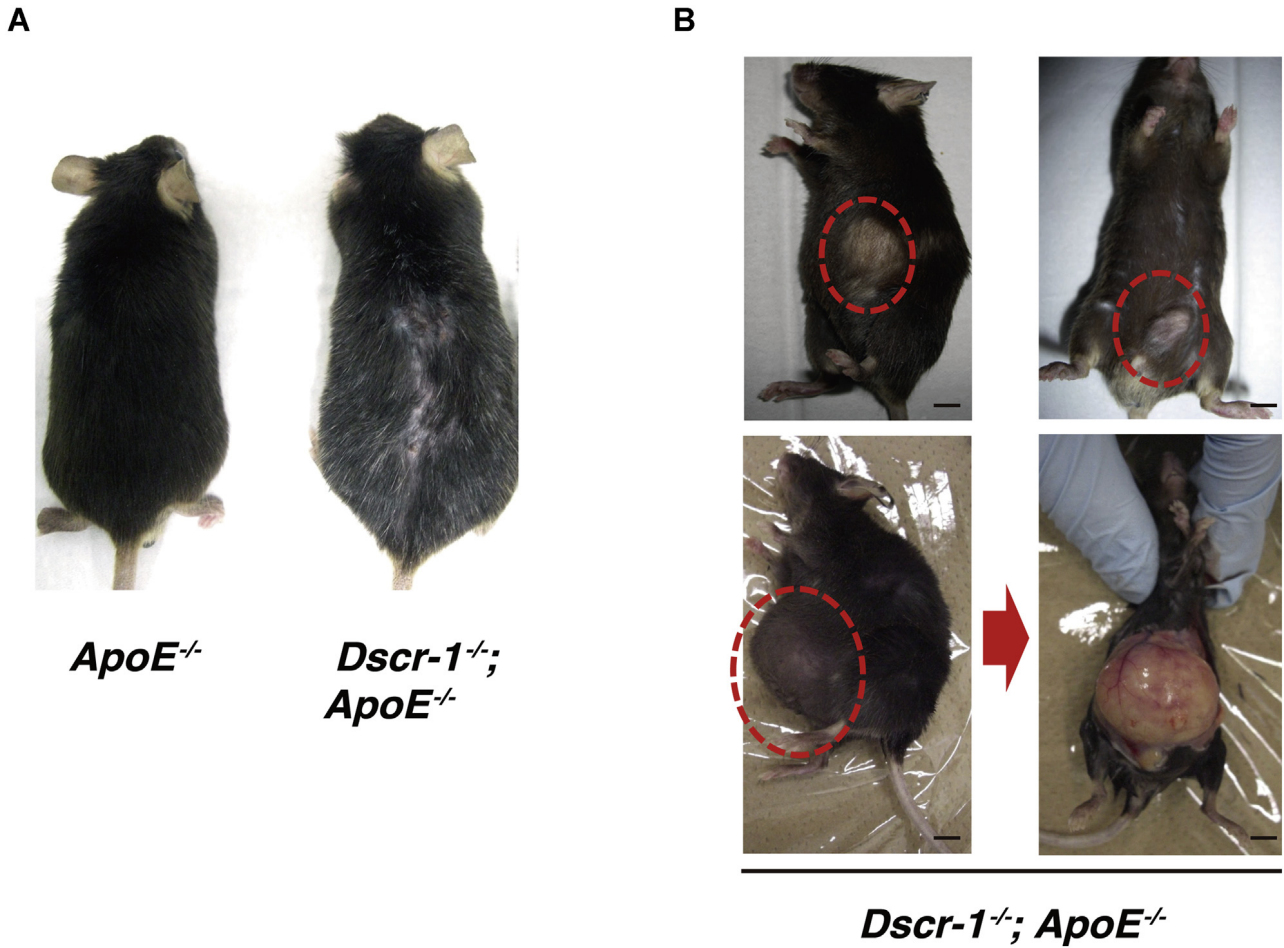
**Dscr-1 deficiency-mediated chronic hypercholesterolemia indicates several pathologies.***A*, representative macroscopic views from 12-month-old mice. Camera distances were ∼15 cm from the subjects. Brightness was autoformatted. *B*, spontaneous lipomas are shown in *red circles*. Scale bar: 1 mm.

In addition to liver failure, cholesterol metabolism can also be influenced by vascular-bed-specific functions, such as lipid absorption into the aortic endothelium. Eighteen-week-old normal chow-fed *ApoE*^−/−^ mice demonstrated atheroma formation compared with WT and Dscr-1^−/−^ mice (Fig. 5*A*). Consistent with previous findings (21), the combined null mutation of *ApoE* and *Dscr-1* failed to induce atheroma compared with the *ApoE*^−/−^ mutation alone (29.8% reduction with *Dscr-1*^−/−^), even though *ApoE* and *Dscr-1* double mutant mice had higher cholesterol profiles (Fig. 5*A*). Impaired migration and accumulation of aortic smooth muscle cells and macrophages for vessel remodeling have been reported in Dscr-1^−/−^ mice (9, 21), leading to resistance to atherosclerotic plaque formation in the aorta. However, the pathology of NFAT signaling pathway mutations has not been fully characterized. To examine whether NFAT/DSCR-1 signaling contributes to the formation of atherosclerotic plaques, we crossed *DSCR-1* promoter-*lacZ*-reporter mice with *ApoE*^−/−^ background, and the resulting mice were stained with anti-lacZ and anti-CD31 antibodies as endothelial markers. In spontaneous atherosclerotic plaques, the *DSCR-1* promoter was specifically activated in the endothelium and smooth muscle cells, as indicated by LacZ expression (Fig. 5, *B*–*D*). This result suggests that Dscr-1 regulates pathological atherosclerosis in the aortic endothelium and smooth muscle cells. To address the role of Dscr-1 in aortic endothelial cells with regard to lipid resorption, we isolated aortic endothelial cells from ApoE^−/−^ alone and *Dscr-1*^*−/−*^ plus *ApoE*^−/−^ mice and performed genome-wide transcriptional analysis (Fig. 6*A* and Fig. S4). Compared with WT and ApoE^−/−^ aortic endothelium, gene expression patterns were similar in Dscr-1^−/−^ mice, except for scavenger receptor A (SR-A) (Fig. S4), whose mRNA was specifically increased only in aortic endothelial cells from ApoE^−/−^ mice but not in WT, Dscr-1^−/−^, or *ApoE* plus *Dscr-1* double null mice (Fig. S4). Importantly, ceruloplasmin mRNAs were specifically and significantly induced in the aorta of Dscr-1^−/−^ null mice, while their expression was not observed in the microvascular endothelium of the lung (Fig. 6*B*). Ceruloplasmin is a copper transporter that leads to nitrite production *via* nitric oxide (NO) (22). It has been reported to function as an antioxidant product in circulation (23). Ceruloplasmin also inhibits endothelial cell NO synthetase activity (24) and can dysregulate NO homeostasis in the aorta. Indeed, free NO levels were reduced by more than threefold in the aorta of *Dscr*-1 null mice (Fig. 6*C*). Furthermore, in an *ex vivo* macrophage adhesion assay in the aorta, pretreatment with oxidative LDL (oxLDL) in the dissected aorta showed marked induction of fluorescently labeled macrophage adhesion in WT mice. In contrast, *Dscr-1* null aortas exhibited significantly reduced cell adhesion to the aortic endothelium (Fig. 6*D*). These data suggest that aortic endothelial cells in Dscr-1^−/−^ mice attenuate the initial step of atherosclerotic plaque formation. Indeed, endothelial-cell-derived activation molecules for macrophages, such as *VCAM-1* and *ICAM-1*, were induced after oxLDL treatment, and these were much weaker in the endothelium of Dscr-1^−/−^ mice than in WT controls (Fig. 6*E*). Consistent with the gene expression profiles in aortic endothelial cells and using WT mice as a reference, *Dscr-1* and *ApoE* double null mice aortas did not accumulate as many lipids compared with *ApoE*^−/−^ mice aortas (Fig. 6*F*). Hypercholesterolemia in peripheral blood was observed in *ApoE* and *Dscr-1* double null mice, whereas increased LDL levels could not be processed by larger aortic vessels due to atherosclerotic plaque formations, leading to worsening higher circulating LDL levels (Fig. 6*F*). Chronic hypercholesterolemia *in vivo* might lead to pathological inflammatory damage, even with less atherosclerosis in large vessels. Compared with ApoE^−/−^ mice, ApoE plus DSCR-1 double null mutated mice 48 weeks old showed uneven hair surfaces and developed spontaneous keloid diathesis and lipomas (Fig. 7). Taken together, these findings show that loss of *Dscr-1* specifically suppresses lipid resorption into the aortic endothelium, resulting in synergistically increased cholesterol levels in peripheral tissue in combination with *ApoE*^−/−^ and *Dscr-1*^*−/−*^-mediated severe liver dysfunction.

## Discussion

Lifestyle diseases, especially hypercholesterolemia, obesity, and chronic inflammation-induced atherosclerosis, are on the increase. Multivascular dysfunction is thought to trigger worse morbidity and mortality. In the present study, we demonstrated that mice with Dscr-1 null mutation exhibited SREBP2 hyperactivation and susceptibility to oxidative or ER stresses and eventually developed NAFLD. Even though Dscr-1^−/−^ alone did not affect the systemic cholesterol clearance, once *ApoE*-null mutation was combined with Dscr-1^−/−^, the mice developed steatohepatitis and a more severe hypercholesterolemia, than *ApoE*-null background alone. Moreover, if such a highly pronounced pathology persisted, they spontaneously developed lipomas with increasing age.

DSCR-1 was named as such because it is located in the Down syndrome critical region of human chromosome 21 (25). We and others have identified that VEGF or thrombin dramatically and rapidly upregulates DSCR-1, adequately modulating calcineurin-NFAT signaling both in primary cultured endothelial cells and *in vivo* (5, 13, 14, 26). Loss of DSCR-1 results in NFAT hyperactivation in endothelial cells, leading to divergent phenotypes due to different tissue microenvironments (17). It has been reported in different mouse lines that *Dscr-1* loss suppressed nonshivering thermogenesis by affecting white adipose tissues and skeletal muscles (27). This metabolic dysfunction may reflect similar conclusions arising from our data, especially related to liver inflammation and fat deposition. However, our generated *Dscr-1* null mutation failed to demonstrate weight loss or feeding insufficiencies compared with littermate WT controls (see Fig. 3, *D* and *E*). Moreover, our isoform-specific *Dscr-1* null mice, recently generated using direct gene-editing technology with C57/BL6J embryos, also revealed normal growth and weight gain, at least under normal chow and no-stress conditions (data not shown). Although the main reasons for the discrepancy in steatohepatitis are still unclear, it may have been caused by intestinal flora or culture conditions. Indeed, pathologies due to *Dscr-1* null mutation were apparent when crossing ApoE^−/−^ mice. Our focus on aortic and liver abnormalities *via Dscr-1* loss would not bear directly on white adipose or skeletal muscles. Most importantly, the calcineurin-NFAT/DSCR-1 signaling axis plays a crucial role in vascular-bed heterogeneity. For example, endothelial cell structure and gene expression can vary in response to the surrounding organ/tissue microenvironment. oxLDL-mediated VCAM-1 or ICAM-1 expression was not fully activated in Dscr-1^−/−^ mice aortic endothelium; however, it was hyper-induced in the lung microvascular endothelium of DSCR-1^−/−^ mice (13). Thus, mice lacking DSCR-1 showed various phenotypes depending on the tissues and organs.

Our data indicated that *Dscr-1* and *ApoE* double null mice experienced significant hypercholesterolemia, while atherosclerotic plaque levels in the aorta were reduced. Among the important players in atherosclerosis, aortic smooth muscle cells and macrophages in Dscr-1^−/−^ mice demonstrated impaired migration and vessel remodeling and promoted M2 macrophage subtypes stimulation, dampening atherosclerotic progression (9). Additionally, conditional deletion of *Dscr-1* predisposes patients to hypertension-mediated intramural hematomas, aneurysms, and aortic rupture (28). These studies indicate that Dscr-1 expression mediates vascular homeostasis through various cellular compartments under aberrant pathological conditions. Here, we show that aortic endothelial cells also largely changed their gene expression profile in Dscr-1^−/−^ mice. Overexpression of ceruloplasmin leads to dysregulation of NO homeostasis. Moreover, SR-A is mainly expressed in macrophages and directly correlates with atherosclerotic progression (29). Although SR-A expression levels in endothelial cells are usually minimal, our microarray analysis indicated significant expression of SR-A mRNAs in aortic endothelial cells in ApoE^−/−^ mice, which was completely lost in mice with both *ApoE* and *Dscr-1* loss (Fig. S4). Further studies are needed to uncover why such plaques failed to grow with hypercholesterolemia in Dscr-1^−/−^ and ApoE^−/−^ mice. Identifying the underlying mechanisms may provide therapeutic targets to prevent plaque progression.

PCSK9 has been identified and linked to the phenotype of familial hypercholesterolemia (30). Studies have uncovered the role of PCSK9 in the regulation of LDLR recycling and identified loss-of-function variants of *PCSK9* associated with lower circulating LDL cholesterol levels and a reduced risk of coronary artery disease (2). Most recently, PCSK9 inhibition also enhanced cytotoxic T cell function with immune-checkpoint therapy against tumors (31). PCSK9 expression is coordinatively regulated by the transcription factors SREBP2 and HNF-1α (32). This is in sharp contrast to LDLR induction, which is directly downstream of SREBP1/2 alone (10). Our data revealed increased LDLR expression in Dscr-1^−/−^ mice livers, but there was no upregulation of endogenous PCSK9 level, although there was an increase in SREBP2. We believe that the main reason for PCSK9 unresponsiveness is a lack of HNF-1α induction, supported by our microarray data (Supplemental data in array, GSE172283).

In conclusion, DSCR-1 is a protective factor against oxidative or ER stress in hepatocytes, chronic liver injuries, metabolic dysfunction, and acute septic inflammation. Loss of *Dscr-1* in the aorta attenuated lipid resorption, which triggered further hypercholesterolemia from ApoE^−/−^ or PCSK9-mediated LDLR knockdown. Our results suggest that the NFAT-DSCR-1 signaling axis is an important regulator of metabolism in the liver and aorta.

## Experimental procedures

The authors declare that all supporting data are available in the article and the Table S1.

### Mice

WT C57BL/6J mice were purchased from Clea Japan. Dscr-1^−/−^ and ApoE^−/−^ mice with C57BL/6J backgrounds have been previously described (17, 33). Hypoxanthine phosphoribosyltransferase (*Hprt*) locus targeted Tie2 promoter-driven human DSCR-1 short isoform expressed mice and DSCR-1 promoter-driven lacZ-expressing mice have also been previously described (13, 18). All animal care and experimental procedures followed instructions from the committee of both the University of Tokyo and Kumamoto University. All animals were allowed free access to water and a normal chow diet (CE-2; CLEA Japan) or a high-fat diet (HFD, ORIENTAL YEAST) containing 35% fat, 30% maltose, 22% casein, 7% dextrin, and 3.5% minerals. Littermate mice were fed HFD for 12 weeks from 8 weeks of age.

### Cell lines

Human embryonic kidney (HEK) 293 cells and HepG2 cells were purchased from the American Type Culture Collection. Cells were maintained in DMEM (Sigma-Aldrich) supplemented with 10% fetal bovine serum (ThermoFisher) at 37 °C in a 5% CO_2_ atmosphere in a humidified incubator. Primary hepatocytes were isolated from mice by enzymatic digestion with collagenase type I and plated on gelatin-coated plates. For induction of lipogenesis, cells starved for 16 h were cultured for 24 h in a medium containing 50 μM Mevastatin (Calbiochem), 50 μM Mevalonolactone (Sigma), 10 μg/ml cholesterol (Sigma), and 1 μg/ml 25-OH-cholesterol (Sigma) in 5% lipoprotein-deprived serum. Primary endothelial cells from the aorta or lungs were isolated and cultured according to a previously described method (18).

### Western blotting and quantification

Liver lysates were prepared with a lysis buffer containing 0.25% NP-40 to extract the cytoplasm and cell membrane proteins. After centrifugation at 4 °C to collect the supernatant, a one-fourth volume of 5x sample buffer was added to the supernatant, and the samples were boiled for 5 min. Aliquots were applied to the gel, and following separation of the proteins by electrophoresis, they were transferred to a membrane and subsequently incubated with 1 μg/ml mouse anti-actin antibody (Sigma) and 1.4 μg/ml rabbit anti-LDL receptor antibody (Abcam) as the primary antibody. Anti-mouse IgG or anti-rabbit IgG antibody conjugated with horseradish peroxidase (HRP) was used as the secondary antibody (GE Healthcare). The obtained images were quantitated using Image J software (NIH).

To separate nuclear and cytoplasmic/membrane fractions, cell lysates were prepared in basal buffer (10 mM HEPES pH 7.6, 150 mM MgCl_2_, 100 mM EDTA, and 100 mM EGTA). To extract cytoplasmic/membrane proteins, basal buffer containing 1 mM KCl was added, and the cells were lysed ten times using a 21-guage needle and then incubated on ice for 30 min. The samples were centrifuged at 1000*g* at 4 °C, and the supernatant was collected. The membrane fraction was concentrated *via* acetone precipitation and treated with lysis buffer (1 mM Tris-HCl pH 7.6, 10 mM NaCl, and 1% SDS). To extract the nuclear fraction, centrifuged precipitates were incubated with basal buffer containing 420 mM NaCl, solubilized, and quantified. Sample aliquots were subjected to SDS-PAGE, the separated proteins were transferred to a membrane and then incubated with antibodies against 1 μg/ml human SREBP2 (provided by the Sakai lab, Tohoku University, Japan), mouse SREBP2 (Abcam), Histone H3 (Cell Signaling Technology), ATF-4 (Santa Cruz Biotech), ATF-6a (Santa Cruz Biotech), or XBP-1S (Santa Cruz Biotech).

### Histological analysis and immunohistochemistry

For paraffin sections, tissues were embedded in paraffin after fixation in 4% paraformaldehyde and subjected to hematoxylin and eosin (H&E) and Oil Red O staining. For cryosection analysis, tissues were sectioned, washed with 60% isopropyl alcohol, and then stained for 1 h at 37 °C in Oil Red O solution (Fujifilm, Wako). Oil Red O-positive lipid droplets were quantified using *Image J* software. For detection of Dscr-1 in the mouse liver, mice were perfused with 2% paraformaldehyde in PBS. Slides containing cryosections were treated with acetone for 10 min, blocked with a protein blocker (DAKO), and stained with our generated anti-Dscr-1 antibody (13). Zenon labeling technology (Invitrogen) was used to amplify the mouse tissue signal using a mouse monoclonal antibody. To detect 4-HNE in mouse liver, tissue slides were incubated with an anti-4-HNE antibody (JaICA) and subsequently with HRP-conjugated secondary antibody; the slides were then incubated with DAB reagent and hematoxylin counterstain.

### *Ex vivo* aortic adhesion assay

WT or Dscr-1^−/−^ mice were anesthetized, aortas resected from the chest cavity were flushed with DMEM containing 0.1% heparin, and extra fat and connective tissue were removed. oxLDL (50 μg/ml) was injected into the aorta and incubated for 4 h at 37 °C. During incubation, peritoneal macrophages activated by thioglycollate medium (BD Difco) were collected from WT mice. Peritoneal macrophages were labeled with PKH26 (Sigma) and injected with 3 × 10^5^ cells through the oxLDL-treated aorta. After 6 h of inoculation, macrophages were washed with Hank’s buffer saline solution (Fujifilm, Wako). The aortas were opened and embedded into a gelatin gel to count the number of adhered macrophages under fluorescent microscopy.

### Quantification of mouse ApoB lipoprotein in serum

Mice were anesthetized and peripheral blood from the inferior vena cava was collected. Serum was obtained by centrifugation for 15 min at 1000*g*, followed by coagulation for 2 h at room temperature. An ELISA (CUSABIO Biotech) was performed according to the manufacturer’s instructions.

### Hypercholesterolemia model generation *via* CA-PCSK9 administration

The recombinant mouse PCSK9 gene was subcloned from mouse liver cDNA and inserted into the Mighty TA-cloning vector (Takara Bio). To obtain CA-mPCSK9 with a D377 to Y mutation, PCR-based point-mutation was performed using specific primers. To produce adeno-associated virus (AAV), we utilized AAV-pro Helper Free System (AAV5) (Takara Bio) and purified it using the AAV-pro Purification Kit (Takara Bio) according to the manufacturer’s protocol. To deliver AAV, 3 × 10^10^ virus genome copies of AAV-CA-mPCSK9 were administered to 10-week-old mice by tail vein injection. Thereafter, mice were fed HFD for 2 weeks, and peripheral blood and liver tissues were collected to examine the serum profile and LDLR expression, respectively. To produce adenovirus, we used the pAd/CMV/V5-DEST Gateway Vector Kit (ThermoFisher) with cloned CA-mPCSK9 according to the manufacturer’s instructions. After infection of HEK293 cells with adenovirus, the culture medium was changed to Pro293, a Serum-Free Medium (LONZA), to collect the CA-mPCSK9 secreted into the medium. The solution was concentrated using a Centriprep (Merck) and infected HEK293 cells were cultured for 3 days, and the resulting adenovirus was amplified. A 5 × 10^9^ pfu virus solution was administered (*i.v.*) to 10-week-old mice. Thereafter, mice were administered with an *i.p.* injection of concentrated CA-mPCSK9 for 2 days. The treated mice were fed HFD until the end of the experiments. Peripheral blood and liver tissues were examined for serum profiling and LDLR expression, respectively. Biochemical profiling of serum components was performed using an automated analyzer (BioMajesty), and LDLR semiquantification was performed based on western blotting.

### Quantification of nitrite and LDL accumulation in the aorta

Nitrite volume in isolated aortas was calculated using the colorimetric assay for NO synthase according to the manufacturer’s protocol (Oxford Biomedical Research) and the methods of Ghigo *et al.* (34). LDL accumulation was quantified using DiI-labeled LDL (Alfa Aesar). Twenty-four-hour fasted mice (8-week-old) were administered (*i.v.*) with 20 μg DiI-LDL. Six hours later, thoracic aortas were isolated and fixed with 3% paraformaldehyde, cryosectioned, and visualized under fluorescent microscopy. Moreover, the unabsorbed LDL level from the plasma was quantified using a colorimetric assay (OD_570_).

### Gene expression analysis using real-time PCR

Total RNA was extracted from tissues using the RNeasy Micro Kit (Qiagen), converted to cDNA using the Prime Script reverse transcriptase (Takara-Bio) according to the manufacturer’s instructions, and used for quantitative real-time PCR amplification using SYBR Green (Takara Bio) with the indicated primers (Table S1).

### Luciferase assay

The functional proximal promoter region of the human SREBP-2 gene was determined *via* enrichment of the histone 3 trimethylated lysine 4 marker using the UCSC genome browser (http://genome.ucsc.edu). The identified region (∼3 kb) was isolated using PCR and subcloned into the pGL4.10 luciferase reporter plasmid (Promega). HEK293 cells were transfected with the SREBP-2 promoter-luciferase vector. After 24 h, cells were treated with CsA or saline control for 30 min and infected with adenovirus containing constitutively active NFAT1 or mock control. Luciferase activity was measured using the Dual-Glo luciferase assay system and normalized to Renilla luciferase following the manufacturer’s instructions (Promega).

### Expression array analysis

For genome-wide transcription analysis, the Affymetrix GeneChip Mouse Genome 430 2.0 Array (Affymetrix) was used. Data were collected and analyzed using the GeneChip Scanner 3000 (Affymetrix) and analyzed using the Affymetrix GeneChip Operating Software v1.3 by MAS5 algorithms to obtain the signal value (GeneChip score) for each probe set. For global normalization, the average signal in the array was set to 100. Expression array data were analyzed using GeneSpring GX software (Tomy Digital Biology). GO analysis was performed using DAVID (https://david.ncifcrf.gov/), and functional annotation was determined by biological processes. GSEA was performed following the standard procedure of the Molecular Signature Database (MSigDB). Transcriptional factor binding motifs commonly shared among characterized gene sets were assessed by MEME and TOMTOM (35).

### Statistical analysis

Data were analyzed using GraphPad Prism 8.0 (GraphPad Software). The normality and variances of data were tested by appropriate tests such as the Kolmogorov–Smirnov test and *F* test and are shown as the mean ± standard deviation. All data passed the normality and equal variance tests. *p*-values between the two groups were calculated using a standard two-tailed Student’s *t*-test. Statistical significance between multiple samples was determined using one-way ANOVA to reveal comparable variance, and each comparison was performed by Tukey HSD post hoc test (Tomy Digital Biology). Statistical significance was set at *p* < 0.05. FDR *q* < 0.25 was considered significant for GSEA analysis.

## Data availability

Expression array data are available from GEO datasets (GSE172283). All remaining data are included in this article.

## Supporting information

This article contains supporting information (14).

## Conflict of interest

The authors have no competing financial interests to disclose.
