# Supplementary material for: Loss of Down syndrome critical region-1 leads to cholesterol metabolic dysfunction that exaggerates hypercholesterolemia in ApoE-null background
Source: J Biol Chem. 2021 Apr 23;296:100697. doi: 10.1016/j.jbc.2021.100697 (PMC8142255; doi:10.1016/j.jbc.2021.100697)
Supplement: Supplemental Figures S1–S4 and Table S1 [file mmc1.pdf]

Table S1

Animals (*in vivo* studies)

Species	Vendor or Source	Background Strain	Sex
Wild type (WT)	Clea Japan	C57BL/6j	M
Dscr-1$^{-/-}$	Ryeom and Folkman Lab	C57BL/6j	M
ApoE$^{-/-}$	Maeda Lab, Taconic, USA	C57BL/6j	M
Dscr-1$^{-/-}$ and *ApoE*$^{-/-}$	Minami Lab	C57BL/6j	M
DSCR-1-lacZ-hprt;ApoE$^{-/-}$	Minami Lab	C57BL/6j	M

Genetically Modified Animals

	Species	Vendor or Source	Background Strain
**Parent – Male and Female**	*Dscr-1*$^{-/-}$	Breeding in our animal facility	C57BL/6j

	Species	Vendor or Source	Background Strain
**Parent – Male and Female**	*ApoE*$^{-/-}$	Breeding in our animal facility	C57BL/6j

	Species	Vendor or Source	Background Strain
**Parent – Male and Female**	*Dscr-1*$^{-/-}$ and *ApoE*$^{-/-}$	Breeding in our animal facility	C57BL/6j

	Species	Vendor or Source	Background Strain
**Parent – Male and Female**	*DSCR-1-lacZ-hprt;ApoE*$^{-/-}$	Breeding in our animal facility	C57BL/6j

Antibodies

Target antigen	Vendor or Source	Catalog #	Working concentration	Persistent ID / URL
CD11b	BD Bioscience	550282	2.5μg/ml	https://www.bdbiosciences.com/ds/pm/tds/550282.pdf
Rat IgG Alexa Fluor 488	ThermoFisher	A21208	2μg/ml	https://www.thermofisher.com/order/genome-database/dataSheetPdf?producttype=antibody&productsubtype=antibody_secondary&productId=A-21208&version=99
LDLR	Abcam	Ab52818	1.4 μg/ml	https://www.abcam.co.jp/ldl-receptor-antibody-ep1553y-ab52818.pdf
Actin	Sigma	A1978	1μg/ml	https://www.sigmaaldrich.com/content/dam/sigma-aldrich/docs/Sigma/Datasheet/6/a1978dat.pdf
Rabbit IgG (H+L)-	ThremoFisher	A11008	2μg/ml	https://www.thermofisher.com/order/genome-database/dataSheetPdf?producttype=antibody&p

Alexa Fluor488				roductsubtype=antibody_secondary&productId=A-11008&version=99
Rat IgG Alexa Fluor 594	ThremoFisher	A21209	2μg/ml	https://www.thermofisher.com/order/genome-database/dataSheetPdf?producttype=antibody&productsubtype=antibody_secondary&productId=A-21209&version=99
Rabbit IgG-HRP	Sigma	A9169	1μg/ml	https://www.sigmaaldrich.com/content/dam/sigma-aldrich/docs/Sigma/Datasheet/7/a9169-074h4802dat.pdf
Mouse IgG-HRP	Sigma	A9044	1μg/ml	https://www.sigmaaldrich.com/content/dam/sigma-aldrich/docs/Sigma/Datasheet/6/a9044dat.pdf
CD31	BD Bioscience	550274	0.078μg/ml	https://www.bdbiosciences.com/ds/pm/tds/550274.pdf
*lac*Z	MBL	PM049	5 μg/ml	https://ruo.mbl.co.jp/bio/dtl/dtlfiles/PM049-v3.pdf
DSCR-1	Institute of Immunology, Co.Ltd, Japan	N2838	5 μg/ml	Originally generated from Minami and Kodama Lab (***J.Biol.Chem.*** 281: 20503-20 2006)
hSREBP2	Institute of Immunology, Co.Ltd, Japan		1 μg/ml	Originally generated from Sakai Lab. (jmsakai@med.tohoku.ac.jp)
mSREBP2	Abcam	ab30682	1 μg/ml	https://www.abcam.com/SREBP2-antibody-ab30682.html

DNA/cDNA Clones

Clone Name	Sequence	Source / Repository
CA-mPCSK9	Primers for mutagenesis<br>Pcsk9-D377Y-FW:<br>TCCAGTTACTGCAGCACATGCTTCATG<br>Pcsk9-D377Y-RV:<br>GCTGCAGTAACTGGACGCTCCGATGAT	Minami lab

siRNA

Clone Name	Sequence	Source / Repository
Si-DSCR-1<br>(targeted both DSCR-1s and DSCR-1L)	5'-CAGAAACUCAGUCUUAUGCAGCUGG-3'<br>5'-CCAGCUGCAUAAGACUAGUUUCUG-3'	Stealth siRNA, Specificity and efficacy was validated from ***J.Biol.Chem.*** 281: 20503-20 (2006)

Cultured Cells

Name	Vendor or Source	Persistent ID / URL
HEK293	ATCC	CRL-1573
HepG2	ATCC	CRL-10741

Data & Code Availability

Description	Source / Repository	Persistent ID / URL
Liver tissue microarray analysis	GSE172283	*https://www.ncbi.nlm.nih.gov/geo/query/acc.cgi?acc=GSE172283*
Aortic endothelial cells microarray analysis	GSE172283	*https://www.ncbi.nlm.nih.gov/geo/query/acc.cgi?acc=GSE172283*

Real-time PCR primers

Primer name	Sequence
Dscr-1-Forward	GACAAACAGTTCCTCATCTCCC
Dscr-1-Reverse	CATCTCTTCCTCTTCCTCCTCA
Ceruloplasmin-Forward	CAACAGTATGTGTATGTGCTGCAT
Ceruloplasmin-Reverse	AGATACCAGCTGAGATTTTCATCC
Msr1-Forward	CTGGACAAACTGGTCCACCT
Msr1-Reverse	TCCCCTTCTCTCCCTTTTGT
Vcam-1-Forward	GAACCCAAACAGAGGCAGAGTG
Vcam-1-Reverse	GGACTGCCCTCCTCTAGTATAGGA
Icam-1-Forward	GGTGACTGAGGAGTTCGACAGAA
Icam-1-Reverse	TCTGCGTCTCCAGGATCTGG
Ppia-Forward	ATCTTGTCCATGGCAAATGCTG
Ppia-Reverse	AAACGCTCCATGGCTTCCAC

Other

Description	Source / Repository	Persistent ID / URL
AAV-pro Helper Free system (AAV5)	Takara Bio	Cat#: 6650
pAd/CMV/V5-DEST Gateway Vector kit	ThermoFisher	Cat#: V49320

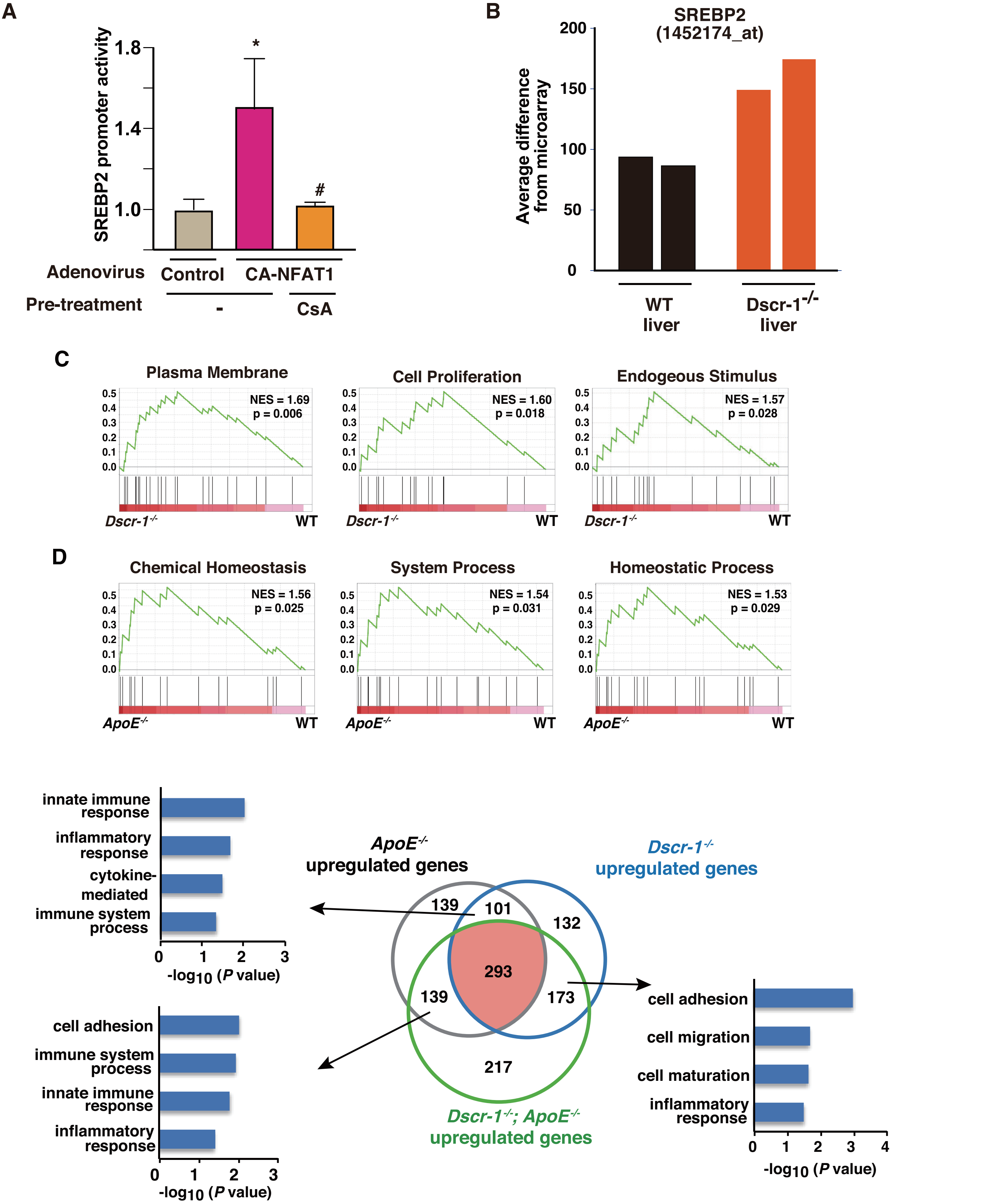

Figure S1 ***Dscr-1*** **null mutation increases SREBP2 and changes cellular homeostasis gene set in liver.**

(**A**) SREBP2-luc activities relative to Ad-control (n=3, mean± SD) *,*P*<0.05 compared with WT, #,*P*<0.05 compared with CA-NFAT in the absence of CsA treatment. (**B**) Average differences of SREBP2 from two independent samples in liver. (**C** and **D**) GSEA pathway analysis of upregulated genes data set from *Dscr-1*$^{-/-}$ (***C***) or *ApoE*$^{-/-}$ (***D***) compared with WT. NES; ApoE, Dscr-1 normalized enrichment score. (**E**) Venn diagrams of more than 2-fold upregulated gene volume in liver from null mutation of either and both of them. GO pathway analysis of commonly upregulated genes data set from *ApoE*$^{-/-}$ and *Dscr-1*$^{-/-}$ (*upper-left*), *ApoE*$^{-/-}$ and *Dscr-1*$^{-/-}$ plus *ApoE*$^{-/-}$ double knockout (*lower-left*), and *Dscr-1*$^{-/-}$ and *Dscr-1*$^{-/-}$ plus *ApoE*$^{-/-}$ knockout (*lower-right*).

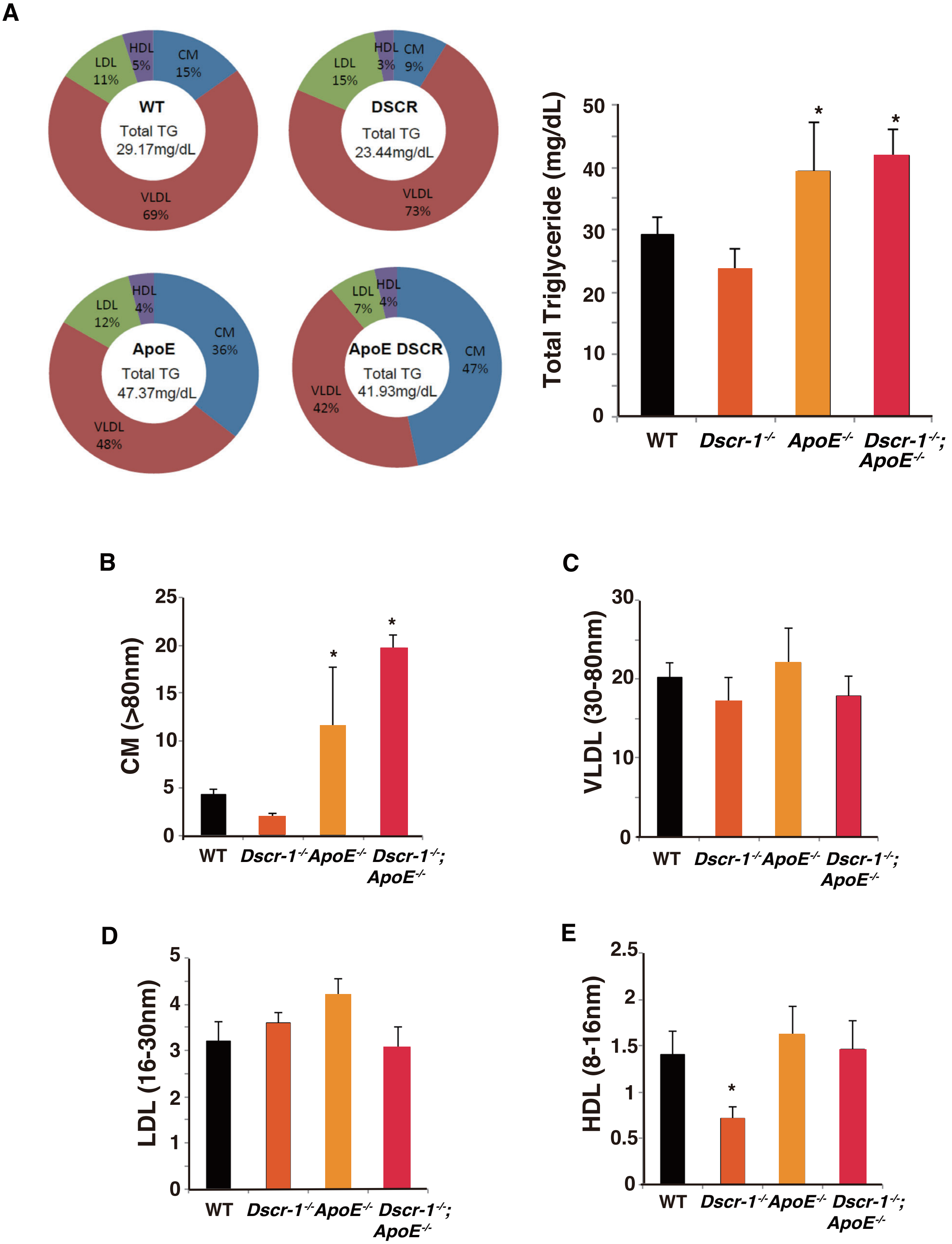

Figure S2 Lipid profiles in Dscr-1, ApoE, and the double null mutation mice.

(**A**) Pie chart of total triglycerides (TG) from either or combined null mutation of Dscr-1 and ApoE, and littermate control (WT) after a high fat diet fed for 8 weeks. (**B-E**). Total TG volume and contents (***B***: chylomicron (CM), ***C***: VLDL, ***D***: LDL, ***E***: HDL) were shown in pie chart. Graph value means total or indicated each content volume (mg/dL) (n=12, mean ± SD) *,*P*<0.05 compared with WT.

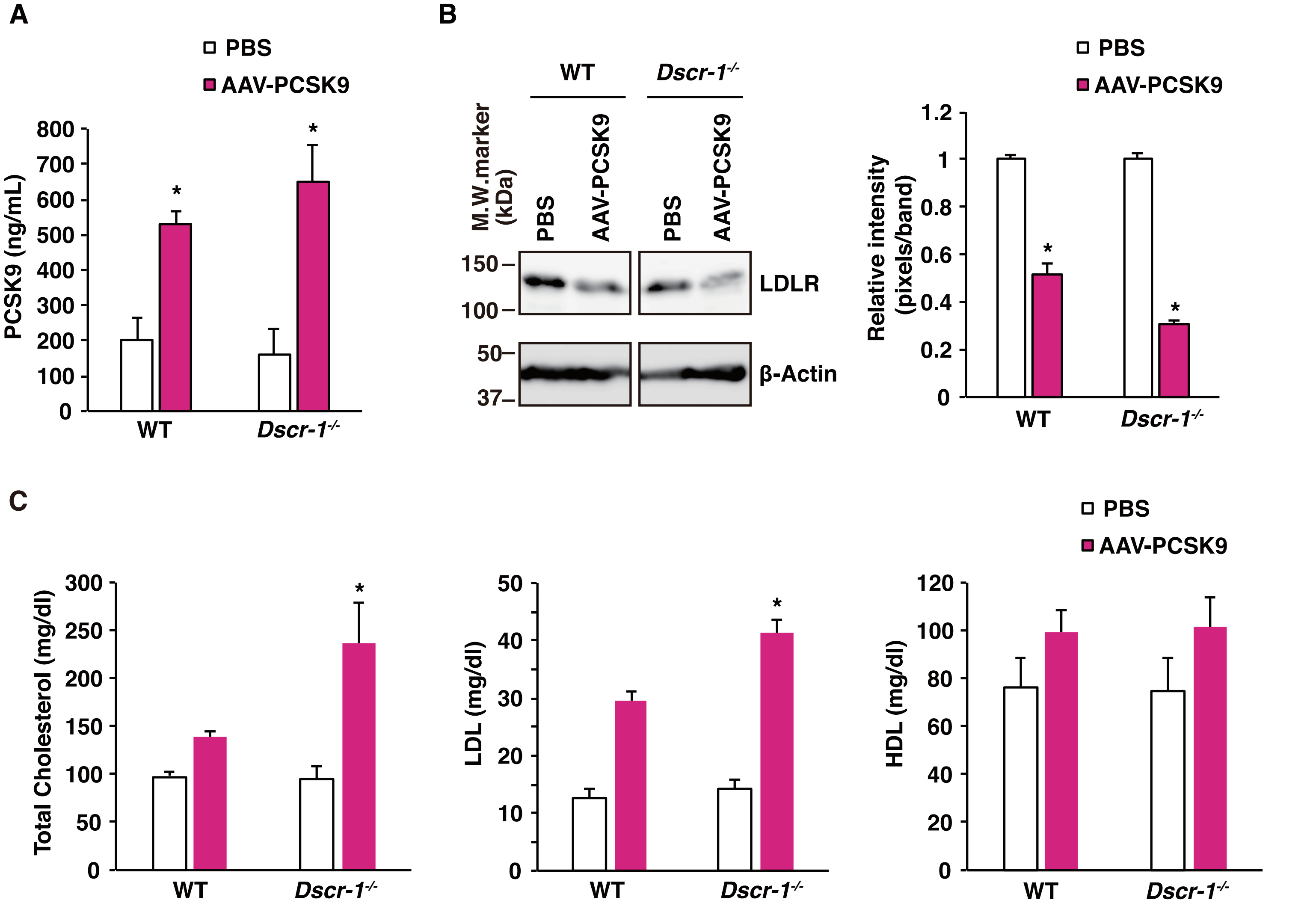

Figure S3 Loss of Dscr-1 exaggerates hypercholesterolemia with adeno-associated virus (AAV)-mediated PCSK9 overexpression.

(**A**) Serum PCSK9 levels at 7 days after the AAV administration. (**B**) Western blot of anti-LDLR antibody from total liver extracts. β-Actin indicates the loading control. *Right* bar graph; LDLR quantification from the western blots (*left*) (n=3, mean± SD) *, $P<0.05$ compared in the mock (PBS) treatment. (**C**) Quantification of total cholesterol (*left*), LDL (*middle*), and HDL (*right*) from indicated conditions (n=3, mean ± SD). *, $P<0.05$ compared with the AAV treatment in WT mice.

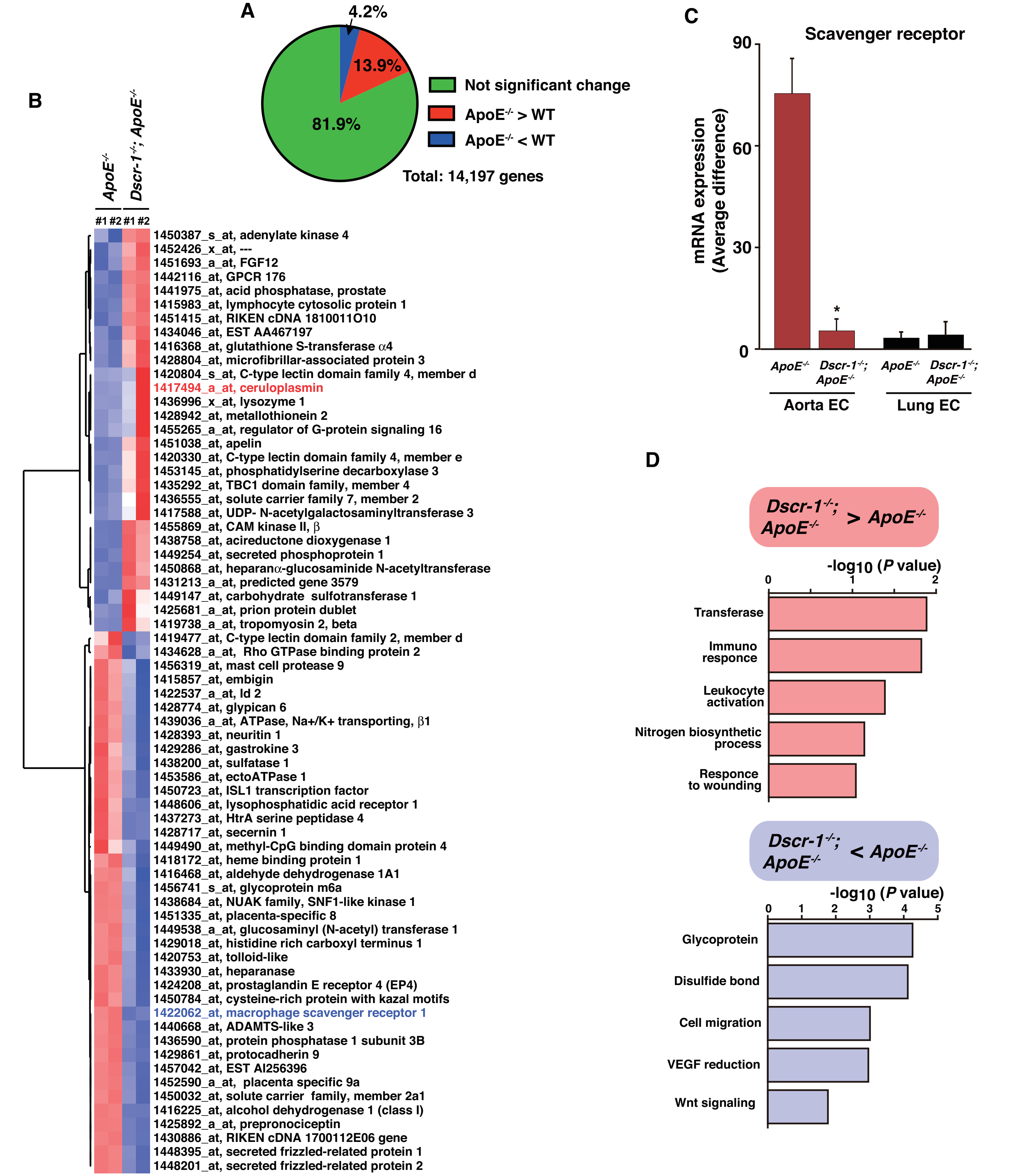

Figure S4 Loss of Dscr-1 in atherosclerotic aorta changes the unique gene expression pattern.

(**A**) Pie chart illustration of changed gene set of aortic endothelial cell (EC)s from *ApoE*$^{-/-}$ and WT. More than two-fold reduced (*blue*), induced (*red*), and not changed (*green*) populations were shown in *ApoE*$^{-/-}$ compared to WT. (**B**) Complete heatmap illustration of more than 2-fold up-or downregulated genes derived from two independent microarrays. (**C**) Average difference of scavenger receptor mRNA expression among *ApoE*$^{-/-}$ and *Dscr-1*$^{-/-}$ plus *ApoE*$^{-/-}$ knockout mice (n=3, mean ± SD) *, P<0.01 compared with *ApoE*$^{-/-}$. (**D**) GO analysis of commonly upregulated (*upper* graph) or downregulated (*lower* graph) gene set in Dscr-1 plus ApoE combined mutation, compared to ApoE single mutation.
